# Role of Endoscopic Retrograde Cholangiography and Nasobiliary Drainage in the Management of Postoperative Biliary Leak

**DOI:** 10.1155/DTE.3.221

**Published:** 1997

**Authors:** M. K. Goenka, R. Kochhar, D. Bhasin, B. Nagi, J. D. Wig, G. Singh, P. V. J. Sriram, K. Singh

**Affiliations:** 1 Department of Gastroenterology and Surgery Postgraduate Institute of Medical Education and Research Chandigarh 160012 India; 2 Eko Endoscopy Center 54 J.L. Nehru Road Calcutta 700071 India

## Abstract

In order to assess the role of endoscopic retrograde cholangiography in evaluating the patients with post-operative biliary leak and of endoscopic nasobiliary drainage in its management, 36 patients with biliary leak seen over a period of 9 years were studied. Thirty-two had biliary leak following cholecystectomy, 3 following repair of liver trauma and 1 following choledochoduodenostomy. Patients presented at an interval of 4 days to 210 days (mean ± SEM, 32.4 ± 6.7 days) following laparotomy. Hyperbilirubinemia was noticed in only 13 patients (36.1%), while abdominal ultrasonogram showed ascites or biloma in 24 (66.7%). Endoscopic retrograde cholangiography showed the leak to involve the common bile duct in 55.6%, cystic duct in 33.3% and intrahepatic biliary radicles in 8.3%. Associated lesions included bile duct obstruction due to stricture or accidental ligature in 20%, bile duct stone in 20% and liver abscess in 2.8%.

Endoscopic nasobiliary drainage using a 7 Fr pig-tail catheter was attempted in 14 patients and could be established in 12 of them. Bile duct leak sealed in all but one of these 12 patients after an interval of 3 days to 40 days (mean ± SEM, 12.2 ± 3.2 days). A single patient with large defect and a proximal bile duct stricture did not respond and required surgery. Common bile duct stones were removed by endoscopic sphincterotomy in 3 out of 4 patients. One patient with large stone required surgical choledocholithotomy. In conclusion, endoscopic retrograde cholangiography was safe and useful in confirming the presence of leak as well as its site, size and associated abnormalities. Endoscopic nasobiliary drainage proved an effective therapy in post-operative biliary leak and could avoid re-exploration in 71.4% patients.


*Diagnostic and Therapeutic Endoscopy*, 1997, Vol. 3, pp. 221–229
Reprints available directly from the publisher
Photocopying permitted by license only

© 1997 OPA (Overseas Publishers Association)
Amsterdam B.V. Published in The Netherlands
by Harwood Academic Publishers
Printed in Singapore

### Role of Endoscopic Retrograde Cholangiography and Nasobiliary Drainage in the Management of Postoperative Biliary Leak

M.K. GOENKA*, R. KOCHHAR, D. BHASIN, B. NAGI, J.D. WIG, G. SINGH, P.V.J. SRIRAM and K. SINGH

*Department of Gastroenterology and Surgery, Postgraduate Institute of Medical Education and Research, Chandigarh – 160012, India*

*(Received 7 February 1996; in final form 25 August 1996)*

**In order to assess the role of endoscopic retrograde cholangiography in evaluating the patients with post-operative biliary leak and of endoscopic nasobiliary drainage in its management, 36 patients with biliary leak seen over a period of 9 years were studied. Thirty-two had biliary leak following cholecystectomy, 3 following repair of liver trauma and 1 following choledochoduodenostomy. Patients presented at an interval of 4 days to 210 days (mean ± SEM, 32.4 ± 6.7 days) following laparotomy. Hyperbilirubinemia was noticed in only 13 patients (36.1%), while abdominal ultrasonogram showed ascites or biloma in 24 (66.7%). Endoscopic retrograde cholangiography showed the leak to involve the common bile duct in 55.6%, cystic duct in 33.3% and intrahepatic biliary radicles in 8.3%. Associated lesions included bile duct obstruction due to stricture or accidental ligature in 20%, bile duct stone in 20% and liver abscess in 2.8%.**

**Endoscopic nasobiliary drainage using a 7 Fr pig-tail catheter was attempted in 14 patients and could be established in 12 of them. Bile duct leak sealed in all but one of these 12 patients after an interval of 3 days to 40 days (mean ± SEM, 12.2 ± 3.2 days). A single patient with large defect and a proximal bile duct stricture did not respond and required surgery. Common bile duct stones were removed by endoscopic sphincterotomy in 3 out of 4 patients. One patient with large stone required surgical choledocholithotomy. In conclusion, endoscopic retrograde cholangiography was safe and useful in confirming the presence of leak as well as its site, size and associated abnormalities. Endoscopic nasobiliary drainage proved an effective therapy in post-operative biliary leak and could avoid re-exploration in 71.4% patients.**

*Keywords:* Common bile duct, endoscopic retrograde cholangiopancreatography, nasobiliary drainage, operative injury

*Correspondence: Dr. M.K. Goenka, Eko Endoscopy Center, 54 J.L. Nehru Road, Calcutta - 700071, India. Fax: 0091-172-2428098; Tel: 0091-172-2428105

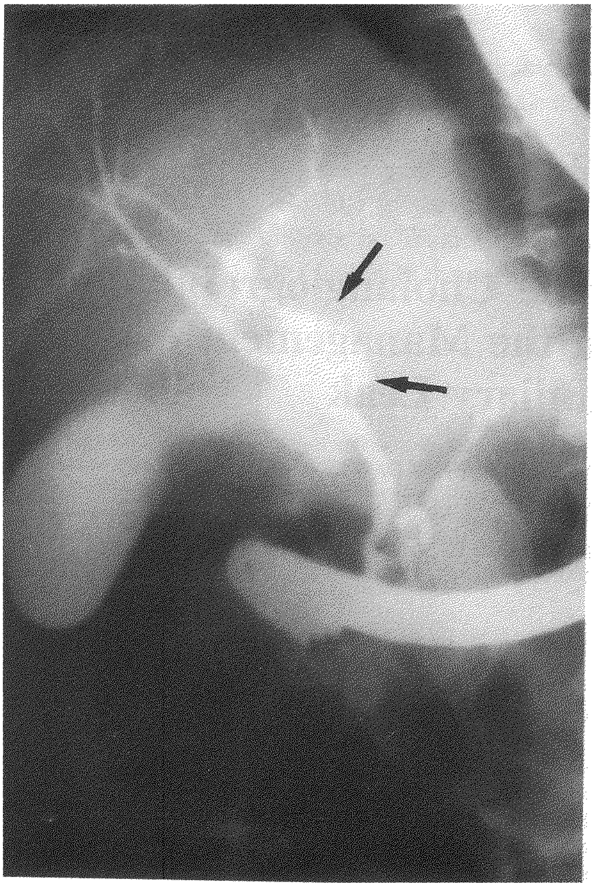

FIGURE 1A ERCP showing leak from intrahepatic biliary radicles (arrows) following laparotomy performed for liver trauma resulting from road-side accident.

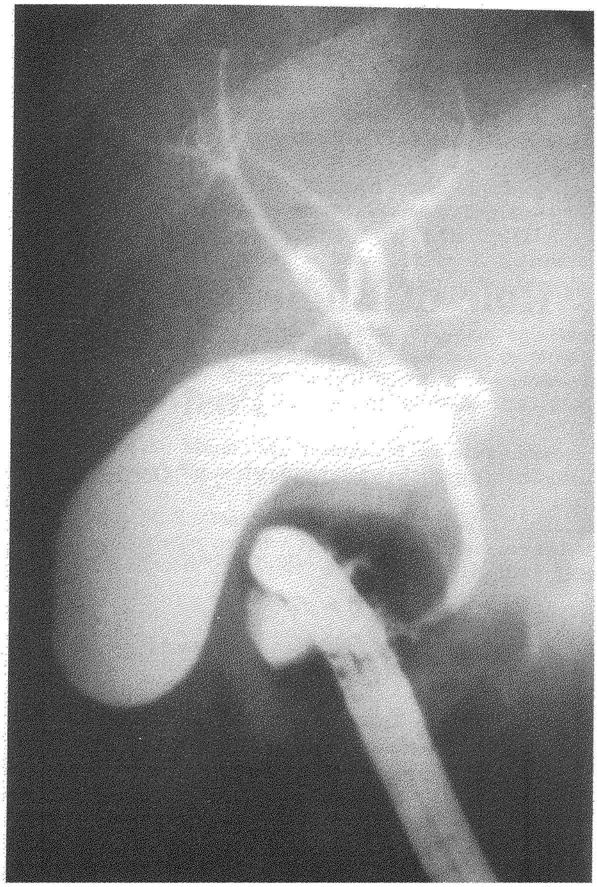

FIGURE 1B Follow-up cholangiogram showing the closure of leak after endoscopic nasobiliary drainage.

### INTRODUCTION

Bile duct injuries including biliary leak complicate open cholecystectomy in about 0.25 to 0.5% cases[1]. The frequency of this complication has gone up to 2.7% with laparoscopic cholecystectomy[2]. In view of a large number of cholecystectomies performed regularly, post-operative biliary injury in spite of a low incidence, comprises a significant clinical problem. Majority of operative bile duct injuries are not recognised during initial surgery and manifest a few days to weeks after laparotomy[3]. Ultrasonogram, percutaneous transhepatic cholangiography, scintigraphy and endoscopic retrograde cholangiopancreatography (ERCP) have been used to diagnose these biliary complications with varying results[4,5].

Biliary leaks have traditionally been treated by surgery[6–8]. Re-exploration however, is often difficult in view of adhesions and inflammation with a reported mortality of 5 to 8%[6–8]. Transhepatic treatment has been shown to be useful but is invasive being associated with considerable risk of bleeding and peritonitis[9,10]. Endotherapy of biliary fistula has been successfully used over the last few years and consists of endoscopic sphincterotomy, biliary stenting and nasobiliary drainage[2,5,11–17]. Available reports have mostly included patients with all types of post-operative biliary problems and since all the three modalities of endotherapy have been used, it is not possible to evaluate the role of one of these endotherapies in selected patients with post-operative biliary leak. We in the present report studied

Table I Spectrum of post-operative biliary abnormalities (n=228)

| | No of patients | % |
|---|---|---|
| Retained/recurrent bile duct stones | 99 | 43.4 |
| Bile duct stricture | 42 | 18.4 |
| Bile duct ligature | 37 | 16.2 |
| Bile duct leaks | 36 | 15.8 |
| Malignant bile duct stricture* | 11 | 4.8 |
| Papillary stenosis | 2 | 0.9 |
| Sclerosing cholangitis | 1 | 0.4 |

*Carcinoma gall-bladder with infiltration of bile duct or cholangiocarcinoma the role of ERCP in evaluating the post-operative biliary fistula and report our experience with endoscopic nasobiliary drainage (ENBD) in its treatment.

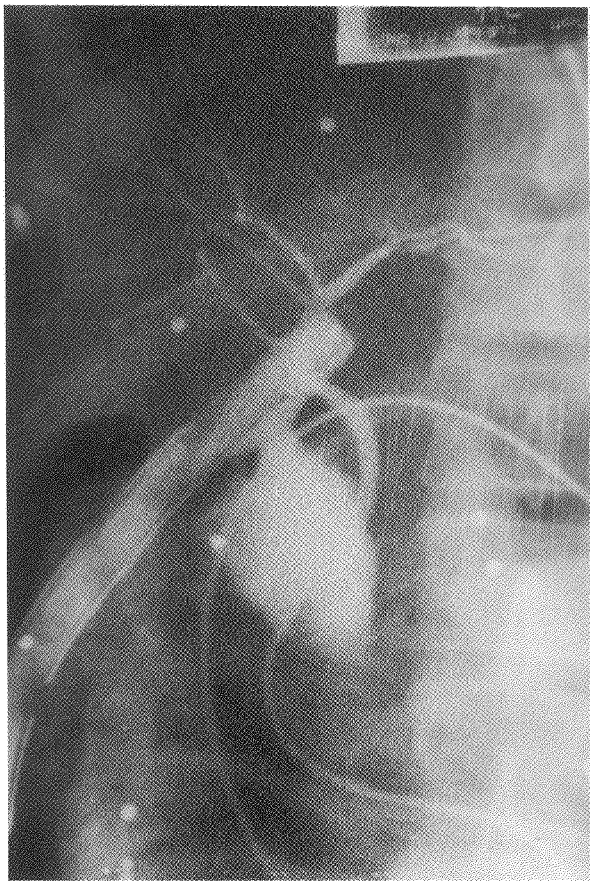

FIGURE 2 Nasobiliary drainage in a patient with biliary leak following cholecystectomy performed for gun shot injury to gall bladder. Scattered pellets and a subhepatic drain are also seen.

### MATERIALS AND METHODS

Over a period of 9 years (Sept '86 to Oct. '95), all patients referred to the Department of Gastroenterology with a diagnosis of post-operative biliary problems were evaluated and those with biliary leak were included for this study. Clinical history and findings were recorded particularly the nature of surgery, its timing and the presenting complaints. All patients underwent laboratory investigations including liver function tests and abdominal ultrasonography was performed in all of them.

A diagnostic ERCP was performed after starting the patient on intravenous antibiotics (Ampicillin + Gentamicin or Ciproflaxacin). Procedure was done under intravenous hyoscine N-butyl bromide (40 mg) with diazepam (5–10 mg) or pentazocine (30 mg) using side viewing duodenoscope (JF $B_2$, 1T, IT-20, Olympus or FD 34 X, Ashai Opticals). Presence of biliary leak was noted and its site, size as well as presence of associated abnormalities like stone, stricture, ligature, cholangitic abscess etc. were recorded. Five patients (all before 1989) also underwent percutaneous transhepatic cholangiography.

While patients till 1992 were managed conservatively or by surgery, those from 1993 onwards were treated by ENBD. ENBD was done in the same sitting as diagnostic ERCP. A 0.035" guide wire (Zebra, Microvasive or Terumo) was passed through the ERCP cannula, cannula was withdrawn and 7 Fr nasobiliary catheter was threaded over the guide wire with an attempt to place the proximal pig-tailed end of the catheter proximal to the site of leak or into the abscess cavity, if present. Simultaneous percutaneous needle or catheter aspiration of associated biloma was carried out under ultrasound guidance. A cholangiogram was obtained through nasobiliary catheter at interval of 3 to 5 days and once the leak was seen to be sealed, catheter was withdrawn after 48 hours. Patients with associated bile duct stones were subsequently subjected to endoscopic sphincterotomy and dormia extraction of stones.

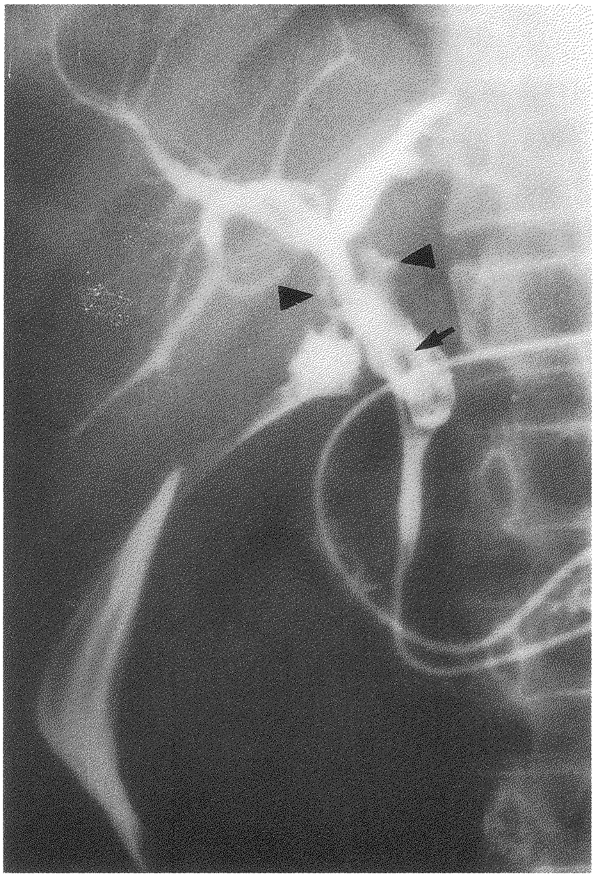

FIGURE 3A Nasobiliary drainage placed in a patient with residual ductal stone (arrow) and a leak from bile duct (arrow heads).

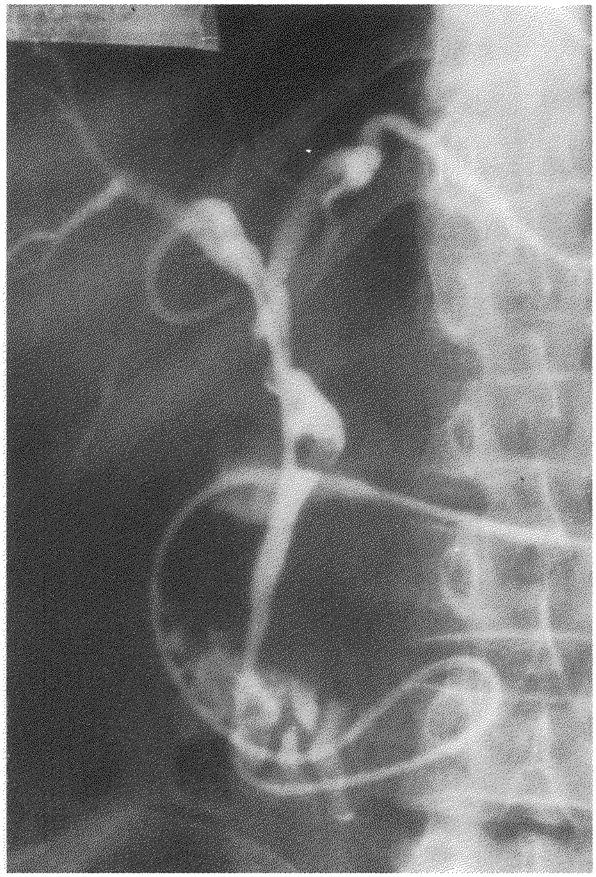

FIGURE 3B Follow-up cholangiogram showing closure of leak. Stones were removed by sphincterotomy subsequently.

### RESULTS

During the study period, a total of 228 patients were diagnosed to have post-operative biliary abnormalities as seen at ERCP (Table I). Biliary leak was diagnosed at ERCP in 36 patients (15.8%). The age of patients with biliary leak ranged from 24 years to 70 years (mean ± SEM, 41.9 ± 2.0); 11 were male and 25 were female. 32 of the 36 patients had biliary leak following cholecystectomy (open: 27, laparoscopic: 5), while 3 had it following surgery for liver trauma; road side accident: 2 (Fig. 1), bullet-injury: 1 and 1 following choledochoduodenostomy performed for idiopathic bile duct stricture. Indications for cholecystectomy included gall stone disease (n=30), carcinoma of gall-bladder (n=1) and gun-shot injury to the gall-bladder (n=1) (Fig. 2).

The time of clinical manifestation after the laparotomy varied from 2 days to 150 days (mean: 15 days, SEM: 5.1 days, median: 7 days), while the patients presented to us after a mean period of 32.4 days (range 4 days to 210 days, median: 20 days, SEM: 6.7 days). Two of these patients had bile duct injury recognised and repaired by end to end anastomosis during initial laparotomy but presented later with leak from the anastomotic site. Clinical presentation of patients with biliary leak included excessive or persistent bile drainage from subhepatic

Table II ERCP findings in patients with biliary leak (n=36)

| | No. of patient | % |
|---|---|---|
| Site of leak | | |
| Common bile duct | 20 | 55.6 |
| Cystic duct | 12 | 33.3 |
| Intrahepatic bile duct | 3 | 8.3 |
| Choledochoduodenostomy site | 1 | 2.8 |
| Associated lesions | | |
| Bile duct stones | 7 | 20.0 |
| Bile duct obstruction* | 7 | 20.0 |
| Liver abscess | 1 | 2.8 |

*Ligation or stricture drain site (n=18), ascites of biliary nature (n=15), jaundice (n=9), cholangitis (n=3) and/or pain abdomen (n=1). Liver function tests revealed a bilirubin level ranging between 0.5 to 12 mg/dl (median: 1.2 mg/dl, mean: 2.8 mg/dl, SEM: 0.74 mg/dl) with hyperbilirubinemia (>2 mg/dl) present in 13 patients (36.1%) only. Abdominal ultrasonography demonstrated evidence of ascites or pericholedochal collection suggestive of biloma in 24 patients (66.7%), while dilated intrahepatic biliary radicles were seen in 7 patients, bile duct stones in 7, liver abscess in 1 and pleural effusion in 1. Of the 5 patients with attempted percutaneous transhepatic cholangiography (PTC), it was successful in 3 (two of them with dilated intrahepatic biliary radicles) and showed extravasation of contrast from biliary tree in all 3 (cystic duct: 2, common bile duct: 1). In other 2 patients with nondilated intrahepatic biliary radicles, PTC was unsuccessful.

Table II summarizes the findings of ERCP which confirmed the leak, showed its site and presence of associated lesions. Common bile duct close to cystic duct stump was the commonest site of leak (Fig. 3). All three patients with leak from intrahepatic ducts had their leak following liver repair done for liver trauma (road side accident: 2, bullet injury: 1), 2 of these had leak onto peritoneal cavity (Fig. 1), while 1 had biliopleural fistula. Size of leak varied from a minute one to about 1 cm.

All the patients till 1992 were managed initially by conservative treatment consisting of repeated needle aspiration or catheter drainage of biloma; 5 had cessation of biliary leak, while 10 required surgical or radiological intervention (hepaticojejunostomy with jejunojejunostomy-4, peroperative T-tube insertion in bile duct-4, percutaneous transhepatic biliary drainage-2). Follow-up was not available in remaining 7 patients. Two patients managed by surgery required post-operative ventilatory support and one of these died of respiratory failure. One patient treated by transhepatic drainage required repositioning of catheter which had slipped out.

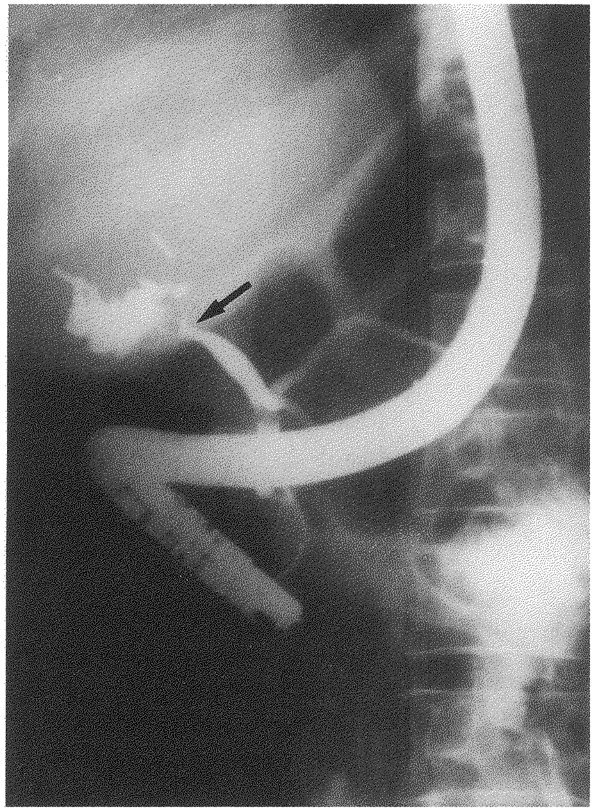

FIGURE 4 Patient with biliary leak following laparoscopic cholecystectomy. Endoscopic drainage failed since guide wire could not be negotiated across the completely transected duct (arrow).

A total of 14 patients were subjected to ENBD, which was successful in 12 patients (Fig. 3), while in 2 it could not be performed because of failure to negotiate the guide-wire across the leak either because of associated bile duct obstruction (1 patient) or because of complete

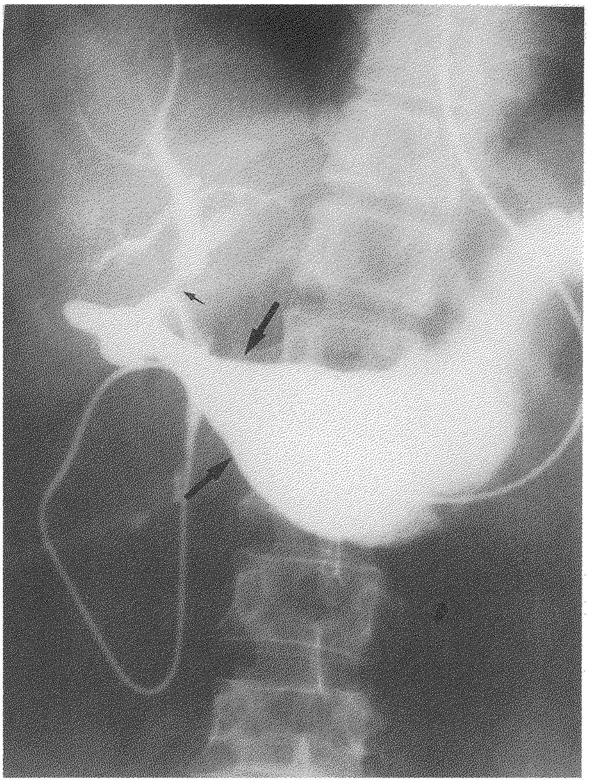

FIGURE 5 Patient with a large biliary leak (thick arrows) and a proximal biliary stricture (thin arrow). Endoscopic nasobiliary drainage failed to seal the leakage.

transection of the bile duct (1 patient; Fig. 4). In 3 patients, ENBD could be placed but the catheter tip could not be positioned proximal to the leak because of peripheral intrahepatic location of the leak (2 patients; Fig. 1) or presence of a stricture proximal to the leak (1 patient; Fig. 5). Biliary leak subsided in 11 out of 12 patients with successful ENBD) after an interval of 3 to 40 days (mean 12.2 d, median 7 d, SEM3.2days) (Fig. 1 and 3). Excluding one patient with associated cholangitic abscess, who required 40 days for the leak to seal (Fig. 6), all other patients had the cessation of leak within 14 days. The single patient who had persistence of biliary leak inspite of ENBD had a large leak (approximately 1 cm) and proximal ductal stricture which could not be negotiated by the ENBD catheter which was placed below the stricture (Fig. 5). This patient required surgery. Four of the 14 patients with attempted ENBD had associated bile duct stones (Fig. 3), 3 of these were removed subsequently with dormia basket following endoscopic sphincterotomy; while one patient with a large stone was subjected to surgery. Surgery was thus required in 4 of these 14 patients, two for failure of ENBD placement and one each for failure of leak to heal in spite of ENBD placement and for a large residual ductal stone.

### DISCUSSION

Excess or persistent bile drainage from subhepatic drain or presence of bile ascites following laparotomy are highly suggestive of iatrogenic biliary leak. These were present in 50% and 41.7% respectively in patients with biliary fistula in the present series. Present study also confirms that liver function test are of no use in predicting the presence of biliary leak. Appropriate management of biliary leak needs proper visualisation of biliary tract anatomy and foreknowledge of its site, size as well as presence of associated lesions. Various investigations used for this purpose include ultrasonogram, cholescintigraphy, PTC and ERCP[4,5,13]. At sonography, fluid collection was seen in 66.7% of our patients. However, sonologically it is difficult to distinguish if the collection is of lymph or bile, loculated ascites or abscess. The visualisation is further obscured by adjacent gas, indwelling T-tube and subhepatic drain[4]. In addition, as expected, ultrasonogram provided no information about site and size of the fistula and was not always helpful in diagnosing associated stone and stricture. Zamel *et al.*[4] had earlier noted a limited role of ultrasonography in evaluating biliary complications following liver transplantation with an overall sensitivity of only 54%. Scintiscan has also proven inadequate with poor sensitivity even in the presence of significant injury of the bile duct and extravasated bile can be wrongly interpreted as bowel loop[18].

PTC was successful in only 3 of the 5 attempted patients; both the patients with unsuccessful PTC had non-dilated biliary radicles. Biliary fistula, because of biliary decompression, is often not associated with dilated intrahepatic biliary radicles making PTC technically difficult[5]. In 3 patients in whom it succeeded, PTC did not add to the information

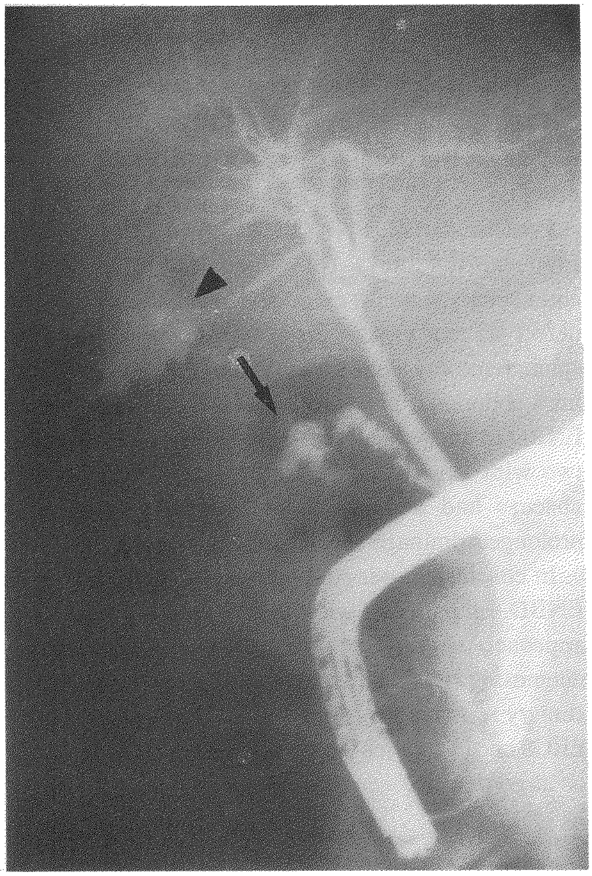

FIGURE 6A Patient with a leak from cystic duct stump (arrow) with a cholangitic abscess (arrow head).

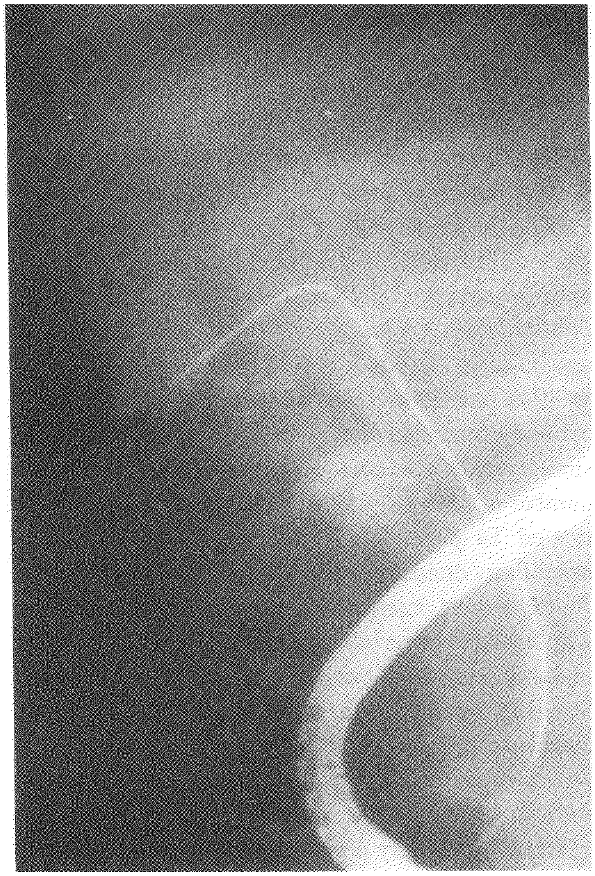

FIGURE 6B Guide wire was passed across the leak into the abscess. Endoscopic nasobiliary drainage could seal the leak with healing of abscess, however this required 40 days of drainage.

provided by ERCP. PTC moreover, is an invasive procedure with considerable risk of bleeding and peritonitis[9,10,19]. ERCP on the other hand was a safe procedure and not only confirmed the leak, but also delineated the exact site and size of the leak as well as of the associated lesions. The usefulness of ERCP in diagnosis of biliary leak has been shown in earlier studies as well[5,13].

Till recently surgery had bee n the mainstay of therapy in accidental lesions of bile duct and included end to end choledochostomy, choledocho/hepaticoenterostomy or simply a per-operative placement of T-tube in the bile duct[6–8]. In our limited experience, two of our patients undergoing re-exploration had significant post-operative morbidity, one of them ultimately succumbed inspite of ventilatory support. Andren-Sandberg *et al.*[7] reported somewhat better results with hepaticojejunostomy compared to end to end choledochostomy, though even with former, good results were obtained in only 54%. Others have also reported a high morbidity, an operative mortality up to 8% and a significant re-operation rate[6,8].

Over the last few years, endotherapy has been successfully used in the treatment of post-cholecystectomy biliary problems. The basic principle of endoscopic therapy in biliary fistula involves reducing the normal 10 mmHg pressure gradient between biliary system and duodenum, the reduced biliary pressure facilitating the closure of fistula[5]. This pressure reduction has been achieved by sphincterotomy, biliary stenting, nasobiliary drainage, stricture dilation and by stone extraction[2,5,12–17].

Most of the available studies on endotherapy of biliary leak have used a variety of techniques[5,13–17], and some of these deal with whole spectrum of biliary abnormalities with only a few cases of biliary leak[2]. Liguory *et al.*[17] treated 52 patients with post-operative fistula, performing endoscopic sphincterotomy in 44 patients, while 8 required endoprosthesis placement, 77% could be treated successfully. Davids *et al.*[14] performed endotherapy in 49 of the 55 patients with biliary leak and could achieve closure of fistula in 43 patients. Somewhat similar results have been reported by others with smaller number of patients[5,12,16]. We, in the present study treated 14 patients with biliary fistula by a 7 Fr nasobiliary drainage and could avoid re-exploration in 71.4% of them. Interestingly leak sealed in two patients with fistula from peripheral intrahepatic radicles inspite of nasobiliary drain being placed distal to fistula site. Stenting or nasobiliary drainage do not act by mechanical sealing of leaking site and hence do not necessarily need to be placed across the defect or in sufficient large diameter to block the fistula[11,13].

While nasobiliary drainage has been used earlier in a limited number of patients[15], most of the experience with endotherapy in biliary leak has been with sphincterotomy or biliary stenting[2,12-14,16,17]. Results of endotherapy with nasobiliary catheter in the present study is similar to that reported earlier with internal biliary stenting[2,12-14,16,17]. We preferred to use a nasobiliary catheter as this permitted a repeated cholangiography to evaluate the time frame of fistula closure and allowed a timely withdrawal of the catheter as well as an early treatment of associated stones. With biliary stents, one is handicapped because the removal at best can be empirical and thus some of the stents may in fact be kept for a period longer than required. Reports in the literature mention biliary stents being kept for 4 months or even longer[5]. In presence of cholangitis, nasobiliary catheter allows collection of bile for culture. One can also obtain an adequate cholangiogram later through the catheter and thus avoid injecting a large amount of contrast initially in an infected biliary system which could aggravate or precipitate septicemia. An obvious disadvantage of ENBD is the discomfort to the patient. However, all but one of our patients required ENBD for less than two weeks and could tolerate the catheter without any problem.

Ponchon *et al.*[5] in their series recorded four favourable factors for successful results with endotherapy namely extrahepatic location of lesion, defect <5 mm in size, distal obstruction treatable by sphincterotomy alone and absence of bile peritonitis or intra-abdominal abscess. We also faced failure with ENBD in one patient with tight biliary stricture and in two with large defects. The patient with cholangitic abscess had a delayed response and required a prolonged drainage for 40 days.

In conclusion, our study confirms good result with ENBD in patients with biliary leak. Endoscopic approach should be the procedure of choice in diagnosing and treating post-operative biliary leak with surgery reserved for patients with anatomy precluding ERCP and in patients with associated difficult strictures or large defects.

### *References*

[1] Moosa, A.R., Mayer, A.D. and Stabile, B. Iatrogenic injuries to the bile duct. *Arch. Surg.* 1990; **125**: 1028–1030.

[2] Manoukian, A.V., Schmalz, M.J., Geenen, J.E., Hogan, W.J., Venu, R.P. and Johnson, K. Endoscopic treatment of problems encountered after cholecystectomy. *Gastrointest. Endosc.* 1993; **39**: 9–14.

[3] Browder, W., Dowling, J.B., Koontz, K.K. and Litwin, M.S. Early management of operative injuries of extrahepatic biliary tract. *Ann. Surg.* 1987; **205**: 649–656.

[4] Zemel, G., Zajko, A.B., Skolnick, M.L., Biron, K.M. and Campbell W.L. The role of sonography and transhepatic cholangiography in diagnosis of biliary complications after liver transplantation. *AJR* 1988; **151**: 943–946.

[5] Ponchon, T., Gallez, J.F., Valette, P.J., Chavaillon, A. and Bory, R. Endoscopic treatment of biliary tract fistulas. *Gastrointest. Endosc.* 1989; **35**: 490–498.

[6] Smith, E.E.J., Bowley, N., Allison, D.J. and Blumgart, L.H. The management of post-traumatic intrahepatic cutaneous biliary fistulas. *Br. J. Surg.* 1982; **69**: 317–318.

[7] Andren-Sandberg, A., Johansson, S., Bengmask, S. Accidental lesions of the common bile duct at cholecystectomy II. Results of treatment. *Ann. Surg.* 1985; **201**: 452–455.

[8] Kune, G.A. and Sali, A. Benign biliary strictures. In: Kune, G.A. and Sai, A., eds. *The practice of biliary surgery.* Boston: Blackwell 1980; 192–244.

[9] von Sonnenberg, E., Casola, G., Wittich G.R. *et al.* The role of interventional radiology for complications of cholecystectomy. *Surgery* 1990; **107**: 632–638.

[10] Trerotola, S.O., Savader, S.J., Lund, G.B. *et al.* Biliary tract complications following laparoscopic cholecystectomy: Imaging and Intervention. *Radiology* 1992; **184**: 195–200.

[11] Brandabur, J.J. and Kozarek, R.A. Endoscopic repair of bile leaks after laparoscopic cholecystectomy. *Semin. Ultrasound. CT MR* 1993; **14**: 375–380.

[12] Kozarek, R.A., Ball, T.J., Patterson, D.J., Brandabur, J.J., Rattz, S. and Traverso ,W. Endoscopic treatment of biliary injury in the era of laparoscopic cholecystectomy. *Gastrointest. Endosc.* 1994; **40**: 10–16.

[13] Kozarek, R.A. Endoscopic techniques in management of biliary tract injuries. *Surg. Clin. North Am.* 1994; **74**: 883–893.

[14] Davids, P.H.P., Rauws, E.A.J., Tytgat, G.N.J. and Huibregtse, K. Postoperative bile leakage: endoscopic management. *Gut* 1992; **33**: 1118–1122.

[15] Burmeister, N., Roppen M.O. and Wurbs D. Treatment of biliocutaneous fistula by endoscopic insertion of a nasobiliary tube. *Gastrointest. Endosc.* 1985; **31**: 279–281.

[16] Foutch, P.G., Harlan, J.R. and Hoefer, M. Endoscopic therapy for patients with a post-operative bile leak. *Gastrointest. Endosc.* 1993; **39**: 416–421.

[17] Liguory, C., Vitale, G.C., Lefebre, J.F., Bonnel, D. and Coronud, F. Endoscopic treatment of postoperative biliary fistulae. *Surgery* 1991; **110**: 779–784.

[18] Meyers, W.C. Interventional radiology in the management of bile duct injuries. *Surg. Clin. North Am.* 1994; **74**; 875–877.

[19] Harbin, W.P., Mueller, P.R. and Ferruci, J.T. Jr. Transhepatic cholangiography. Complications and use patterns of fine needle technique – a multi-institutional survey. *Radiology* 1980; **135**: 15–22.

